# Scrub Typhus and Dengue Co-infection in an Adolescent Girl: A Diagnostic Challenge

**DOI:** 10.7759/cureus.40810

**Published:** 2023-06-22

**Authors:** Rajesh Kanna Kannabiran, Arjun Banerjee, Lakshmi Jyothi, Mounika Reddy, Rahul Narang

**Affiliations:** 1 Department of Microbiology, All India Institute of Medical Sciences, Bibinagar, Hyderabad, IND; 2 Department of Pediatrics, All India Institute of Medical Sciences, Bibinagar, Hyderabad, IND

**Keywords:** tropical infection, acute undifferentiated febrile illness, coinfection, dengue, scrub typhus

## Abstract

Scrub typhus and dengue fever are common infectious diseases in tropical regions, and both have overlapping clinico-epidemiological and laboratory features, which often pose a diagnostic challenge. This case report discusses a 15-year-old girl from the Indian subcontinent who presented with acute undifferentiated febrile illness (AUFI) without typical features of any of the common tropical infections. She was diagnosed with co-infection of scrub typhus and dengue fever using laboratory tests with good diagnostic accuracy. The patient was managed on an ambulatory basis, treated with oral doxycycline, and showed symptomatic improvement within 48 hours. Co-infections in endemic areas present a significant diagnostic and therapeutic challenge. This case report highlights the importance of considering co-infections in the differential diagnosis of AUFI, especially during the post-monsoon period, and the use of highly sensitive and specific tests for the diagnosis of co-infections.

## Introduction

Scrub typhus and dengue are common infectious diseases in tropical regions, including the Indian subcontinent. They share similar clinico-epidemiological and laboratory features such as fever, rash, thrombocytopenia, and hepatic dysfunction. Both involve similar underlying pathophysiological mechanisms including endotheliopathy, capillary leak, and third spacing. Scrub typhus has been reported to have a community seroprevalence of 34.2% in India, and is responsible for 25.3% of cases of acute undifferentiated febrile illness (AUFI), with a high incidence of multiple organ dysfunction (17.4%) and case fatality (6.3%) [1].

Dengue seroprevalence in the general population and case fatality rate among laboratory-confirmed patients has been reported to be 56.9% and 2.6%, respectively, and the prevalence of laboratory-confirmed dengue infection among clinically suspected patients is 38.3% [2]. Ahmed et al. reported a 16% prevalence of various co-infections in hospitalized patients with AUFI in North India [3]. While co-infections are rarely reported, they pose a significant challenge in diagnosis and management. In this report, we discuss the case of an adolescent girl with co-infection of scrub typhus and dengue, who presented without any typical features of either infection and was managed on an ambulatory basis with an uneventful recovery. We highlight the importance of considering co-infections in endemic areas, especially in the post-monsoon season, and the use of sensitive and specific tests for diagnosis.

## Case presentation

A 15-year-old girl presented, in the post-monsoon season, with a high-grade fever of 13 days duration with multiple daily spikes, associated with chills, myalgia, malaise, and decreased appetite. She also gave a history of pain in the left knee for three days, especially while walking. There was no history of rash, cough, breathlessness, vomiting, loose stools, pain abdomen, jaundice, burning micturition, decreased urine output, seizures, or bleeding manifestations. There was no history of recent travel or any febrile illness in the past six months. She was moderately nourished and well-oriented. At presentation, she recorded a temperature of 98.5°F, pulse rate of 141 beats/minute, and blood pressure of 90/60 mmHg. The patient had tachycardia, but there were no other features of shock. Blood pressure was appropriate for age, and peripheral perfusion was good. On examination, there was no pallor, icterus, lymphadenopathy, oedema, rash, or eschar. The abdomen was soft and non-tender, and no organomegaly was noted. There was no local rise in temperature, erythema, tenderness, or restricted movements of the left knee. Other systemic examinations revealed no abnormality.

With a syndromic diagnosis of AUFI, infectious causes were considered most likely; she was investigated for all the common tropical infections prevalent in the region like dengue, scrub typhus, malaria, and enteric fever [4]. Non-infectious causes like rheumatological conditions or malignancies were other possibilities considered. On evaluation, she was found to have microcytic hypochromic anaemia (haemoglobin 10.8g/dL, mean corpuscular volume (MCV) 67.9µm, mean corpuscular haemoglobin (MCH) 21.6pg, mean corpuscular haemoglobin concentration (MCHC) 31.8g/dL), with normal platelet (201,000/µL) and leukocyte (4750/µL; neutrophil 69.4%, lymphocytes 23.3%) counts. C-reactive protein was elevated (96 mg/L) (Table 1).

**Table 1:** Haematological investigations of the index patient RBC: red blood cell; MCV: mean corpuscular volume; MCH: mean corpuscular haemoglobin; MCHC: mean corpuscular haemoglobin concentration; WBC: white blood cell

Haematological investigations	Values	Reference range
RBC count	5.01 × 10^8^/µL	3.80-5.20
Haemoglobin	10.8g/dL	11.5-15.2
Haematocrit	34.0%	35.0-46.0
MCV	67.9µm	77.0-97.0
MCH	21.6pg	26.0-34.0
MCHC	31.8g/dL	32.0-35.0
Platelet count	201×10^3^/µL	150-400
WBC count	4.75×10^3^/µL	3.50-10.0
Neutrophils	69.4%	40.0-73.0
Lymphocytes	23.3%	18.0-45.0
Monocytes	6.6%	4.0-12.0
Eosinophils	0.2%	0.5-7.0
Basophils	0.5%	0.0-2.0

She was found to be positive for scrub typhus IgM and dengue IgM antibodies on a rapid immunochromatography test (ICT). Scrub typhus IgM (Scrub Typhus IgM Microlisa, J. Mitra & Co. Pvt. Ltd, New Delhi, India; sensitivity 100% and specificity 98.6%) [5] and dengue IgM (Dengue IgM Capture ELISA kit, National Institute of Virology, Pune, India; sensitivity 98.5% and specificity 98.8%) [6] confirmed the presence of these infections. Dengue NS1 antigen and IgG antibodies test, Widal agglutination test for *Salmonella*, and malarial *Plasmodium falciparum*/*Plasmodium vivax* (Ps/Pv) antigen test were negative, and there were no malarial parasites detected on a peripheral blood smear (Table 2). We further confirmed the dengue serotype as DEN-2 by RealStar® Dengue Type RT-PCR Kit 1.0 (altona Diagnostics GmbH, Hamburg, Germany) [7]. Based on these reports, a diagnosis of scrub typhus and dengue co-infection was made. She was managed on ambulatory basis as she did not have any complications or organ dysfunctions. Oral doxycycline was initiated for scrub typhus, along with adequate hydration and symptomatic treatment. She showed improvement within 48 hours and recovered without any complications with a seven-day course of doxycycline.

**Table 2:** Results of serological investigations ICT: immunochromatography test; ELISA: enzyme-linked immunosorbent assay; RT-PCR: reverse transcription-polymerase chain reaction; Ig: immunoglobulin; Pf: *Plasmodium falciparum; *Pv: *Plasmodium vivax*

Test	Method	Result
C-reactive protein	Latex agglutination	Positive (96mg/L)
Malarial antigen Pf/Pv	ICT	Negative
Widal Test	Agglutination	Negative SO, SH, AH, BH < 1:20
Scrub typhus IgM/IgG antibodies	ICT	IgM: Positive
Scrub typhus IgM antibodies	Indirect ELISA	Positive (Scrub typhus IgM Units = 83.75 , Scrub typhus IgM Units > 11 = positive)
Dengue NS1 antigen and IgM/IgG antibodies	ICT	IgM: Positive, NS1: Negative, IgG: Negative
Dengue IgM antibodies	IgM capture ELISA	Positive (Patient OD Value = 1.96 , Sample OD > 0.81= positive)
Dengue Serotyping	RTPCR	DEN-2 serotype

## Discussion

Scrub typhus and dengue are both common causes of AUFI (fever of less than two weeks duration without any localizing features of infection) in tropical regions like India. Both are vector-borne diseases with peak incidence during the post-monsoon period. Scrub typhus is a rickettsial infection transmitted by trombiculid mites, while dengue is a viral infection transmitted by *Aedes aegypti* mosquitoes. They often present a diagnostic challenge due to overlapping clinical and laboratory features, including fever, rash, oedema, thrombocytopenia, and hepatic dysfunction [1,2]. While concurrent infections with multiple pathogens may be common in the tropics, co-infections of scrub typhus and dengue are not frequently reported, likely due to differences in vectors, their breeding habits, and biting behaviours. Nonetheless, the increasing incidence of scrub typhus in all regions of India emphasizes the importance of considering co-infections in the differential diagnosis of AUFI, especially during the post-monsoon period.

Concurrent infections with multiple pathogens may have an atypical presentation or protracted course, making diagnosis and management difficult. Previous studies have reported conflicting results regarding the severity of co-infections compared to mono-infections. Basheer et al. reported six cases of scrub typhus and dengue co-infections in adults from South India, with greater tachycardia, hypotension, thrombocytopenia, transaminitis, hypoalbuminemia, and lengthier hospital stay compared to either infection alone [8]. However, Ahmed et al. reported that co-infections were associated with milder clinical manifestations, organ dysfunction, and severity compared to mono-infections [3]. Jose et al. from South India reported scrub typhus co-infection in 51 of 606 dengue patients aged 0-14 years [9]. However, all these were retrospective studies and did not consider the sensitivity and specificity of the diagnostic tests used and possible serological cross-reactivity.

Despite presenting with AUFI during the post-monsoon period, our patient did not exhibit typical features of scrub typhus or dengue infection, including rash, oedema, eschar (dark scab-like region at the site of chigger bite), thrombocytopenia, or transaminitis. However, tachycardia was observed, which was disproportionate to the temperature. The co-infection was confirmed using highly sensitive and specific tests, leaving no doubt in the diagnosis. The scrub typhus IgM micro enzyme-linked immunoassay (ELISA) used in this case is known to have possible serological cross-reactivity with typhoid fever [5], however, it was ruled out by a negative Widal test. Dengue IgM ELISA kit does not have any serological cross-reactivity [6]. Most of the previous reports of scrub typhus and dengue co-infections were in patients who were critically-ill or had multi-organ dysfunction, and serological cross-reactivity could not be ruled out in the earlier reports. Our patient did not have any complications or organ dysfunctions in spite of the co-infection. She was managed on an ambulatory bases and the initiation of appropriate antimicrobial therapy likely contributed to the uneventful recovery of the patient.

Co-infections of scrub typhus and dengue can pose diagnostic and therapeutic challenges in endemic areas. Our case highlights the importance of maintaining a high index of suspicion and conducting a comprehensive evaluation of patients with AUFI, particularly during the post-monsoon period when the incidence of vector-borne diseases is high. It is possible that co-infections are more prevalent than reported and are often overlooked. Therefore, searching for multiple etiologies should be a part of the initial routine diagnostic workup for patients with AUFIs [4]. The accuracy of diagnostic tests used, as well as the potential for serological false-positivity with cross-reacting and pre-existing antibodies, should also be taken into account. Clinicians must be familiar with the complexities involved in diagnosing and managing such cases.

## Conclusions

Despite being infected by both scrub typhus and dengue, the 15-year-old patient made an uneventful recovery after the initiation of doxycycline, based on laboratory investigations. Co-infection of scrub typhus and dengue is an uncommon but possible cause of AUFI in tropical countries like India, especially in the post-monsoon season. Overlaps in clinical presentation and seasonality pose a serious challenge to clinicians in correctly diagnosing and managing such co-infections. Therefore, a high index of suspicion, proper history taking, thorough physical examination, and use of highly sensitive and specific laboratory tests are important for diagnosis.
